# Supplementary material for: Surface Microbiome Profiling of Dental Elevators Using Third‐Generation Sequencing: Implications for Infection Control in Dental Practice
Source: Microbiologyopen. 2025 Dec 8;14(6):e70178. doi: 10.1002/mbo3.70178 (PMC12685768; doi:10.1002/mbo3.70178)
Supplement: Supplementary file 1 — customer_backup. [file MBO3-14-e70178-s001.zip › customer_backup/customer_backup/taxa_summary/Phylogenetic_tree/allsample/ggtreeExtra.abundance.pdf]

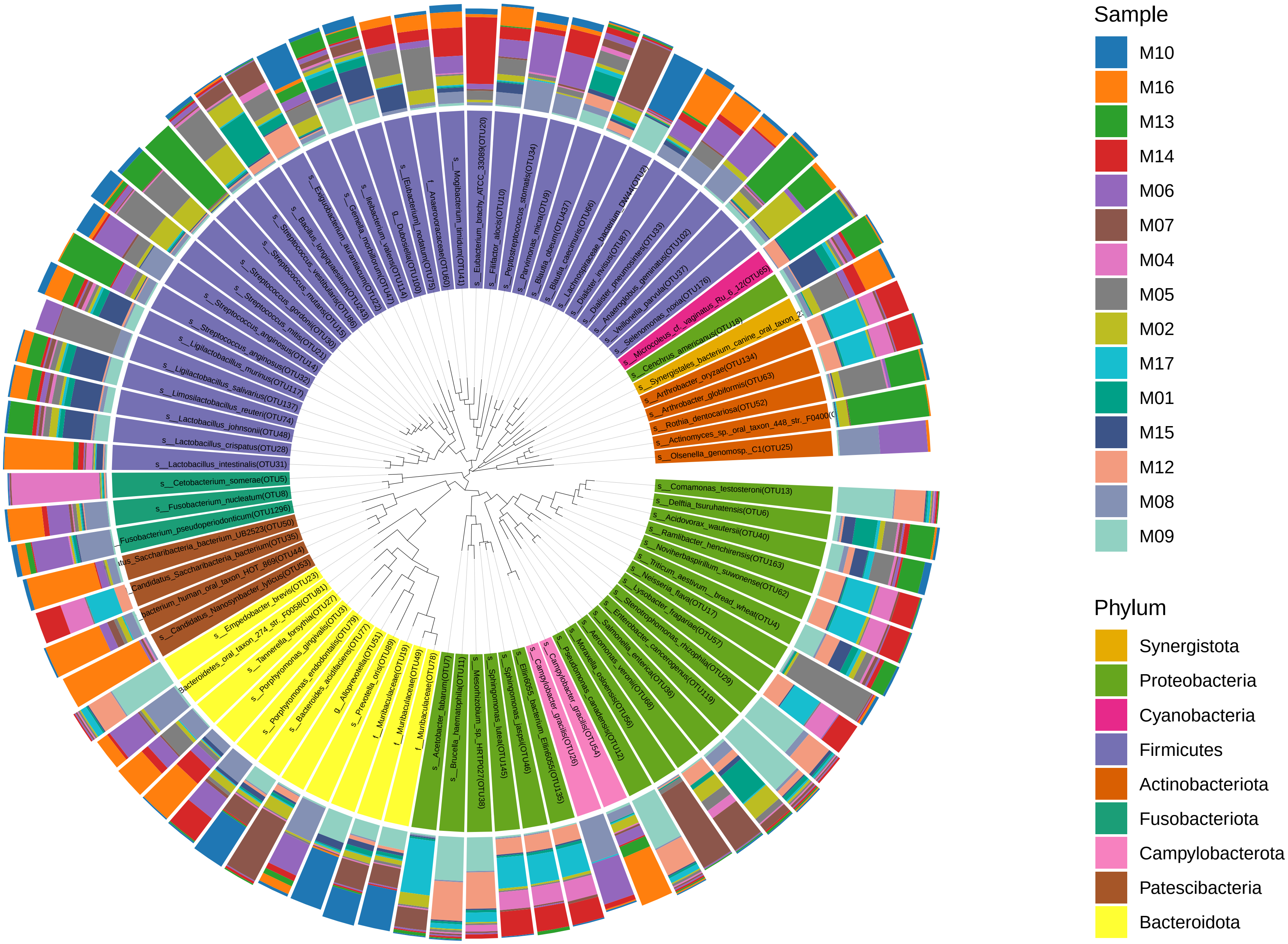
Sample
M10
M16
M13
M14
M06
M07
M04
M05
M02
M17
M01
M15
M12
M08
M09
Phylum
Synergistota
Proteobacteria
Cyanobacteria
Firmicutes
Actinobacteriota
Fusobacteriota
Campylobacterota
Patescibacteria
Bacteroidota
s__Comamonas_testosteroni(OTU13)
s__Delftia_tsuruhatensis(OTU6)
s__Acidovorax_wautersii(OTU40)
s__Ramlibacter_henchirensis(OTU163)
s__Noviherbaspirillum_suwonense(OTU62)
s__Triticum_aestivum__bread_wheat(OTU4)
s__Neisseria_flava(OTU17)
s__Lysobacter_fragariae(OTU57)
s__Stenotrophomonas_rhizophila(OTU29)
s__Enterobacter_cancerogenus(OTU119)
s__Salmonella_enterica(OTU36)
s__Aeromonas_veronii(OTU68)
s__Moraxella_osloensis(OTU56)
s__Pseudomonas_canadensis(OTU12)
s__Campylobacter_gracilis(OTU54)
s__Campylobacter_gracilis(OTU26)
s__Ellin6055_bacterium_Ellin6055(OTU135)
s__Sphingomonas_jaspsi(OTU46)
s__Sphingomonas_lutea(OTU145)
s__Mesorhizobium_sp._HRTP027(OTU38)
s__Brucella_haematophila(OTU11)
s__Acetobacter_fabarum(OTU7)
f__Muribaculaceae(OTU78)
f__Muribaculaceae(OTU49)
f__Muribaculaceae(OTU19)
s__Prevotella_oris(OTU89)
g__Alloprevotella(OTU51)
s__Bacteroides_acidifaciens(OTU77)
s__Porphyromonas_endodontalis(OTU79)
s__Porphyromonas_gingivalis(OTU3)
s__Tannerella_forsythia(OTU27)
Bacteroidetes_oral_taxon_274_str._F0058(OTU81)
s__Empedobacter_brevis(OTU23)
s__Candidatus_Nanosynbacter_lyticus(OTU53)
bacterium_human_oral_taxon_HOT_869(OTU44)
Candidatus_Saccharibacteria_bacterium(OTU35)
atus_Saccharibacteria_bacterium_UB2523(OTU50)
Fusobacterium_pseudoperiodonticum(OTU1296)
s__Fusobacterium_nucleatum(OTU8)
s__Cetobacterium_somerae(OTU5)
s__Lactobacillus_intestinalis(OTU31)
s__Lactobacillus_crispatus(OTU28)
s__Lactobacillus_johnsonii(OTU48)
s__Limosilactobacillus_reuteri(OTU74)
s__Ligilactobacillus_salivarius(OTU137)
s__Ligilactobacillus_murinus(OTU117)
s__Streptococcus_anginosus(OTU32)
s__Streptococcus_anginosus(OTU14)
s__Streptococcus_mitis(OTU21)
s__Streptococcus_gordonii(OTU30)
s__Streptococcus_mutans(OTU15)
s__Streptococcus_vestibularis(OTU86)
s__Bacillus_longiquaesitum(OTU243)
s__Exiguobacterium_aurantiacum(OTU22)
s__Gemella_morbillorum(OTU47)
s__Ileibacterium_valens(OTU114)
g__Dubosiella(OTU100)
s__[Eubacterium]_nodatum(OTU75)
f__Anaerovoracaceae(OTU60)
s__Mogibacterium_timidum(OTU41)
s__Eubacterium_brachy_ATCC_33089(OTU20)
s__Filifactor_alocis(OTU10)
s__Peptostreptococcus_stomatis(OTU34)
s__Parvimonas_micra(OTU9)
s__Blautia_obeum(OTU437)
s__Blautia_caecimuris(OTU66)
s__Lachnospiraceae_bacterium_DW44(OTU2)
s__Dialister_invisus(OTU87)
s__Dialister_pneumosintes(OTU33)
s__Anaeroglobus_geminatus(OTU102)
s__Veillonella_parvula(OTU37)
s__Selenomonas_noxia(OTU176)
s__Microcoleus_cf._vaginatus_Ru_6_12(OTU65)
s__Cenchrus_americanus(OTU18)
s__Synergistales_bacterium_canine_oral_taxon_2
s__Arthrobacter_oryzae(OTU134)
s__Arthrobacter_globiformis(OTU63)
s__Rothia_dentocariosa(OTU52)
s__Actinomyces_sp._oral_taxon_448_str._F0400(O
s__Olsenella_genomosp._C1(OTU25)
